# Scenario analysis of COVID-19 dynamical variations by different social environmental factors: a case study in Xinjiang

**DOI:** 10.3389/fpubh.2024.1297007

**Published:** 2024-02-16

**Authors:** Ruonan Fu, Wanli Liu, Senlu Wang, Jun Zhao, Qianqian Cui, Zengyun Hu, Ling Zhang, Fenghan Wang

**Affiliations:** ^1^School of Public Health, Xinjiang Medical University, Urumqi, Xinjiang, China; ^2^Center of Disease Control and Prevention of Xinjiang Uygur Autonomous Region, Urumqi, Xinjiang, China; ^3^School of Mathematics and Statistics, Ningxia University, Yingchuan, Ningxia, China; ^4^School of Global Health, Shanghai Jiao Tong University School of Medicine, Shanghai, China; ^5^State Key Laboratory of Desert and Oasis Ecology, Xinjiang Institute of Ecology and Geography, Chinese Academy of Sciences, Urumqi, Xinjiang, China; ^6^Daizhuang Hospital, Jining, Shandong, China

**Keywords:** COVID-19 pandemic, Xinjiang Uygur Autonomous Region, social environmental factors, simulation and prediction, scenarios analysis

## Abstract

**Background:**

With the rapid advancement of the One Health approach, the transmission of human infectious diseases is generally related to environmental and animal health. Coronavirus disease (COVID-19) has been largely impacted by environmental factors regionally and globally and has significantly disrupted human society, especially in low-income regions that border many countries. However, few research studies have explored the impact of environmental factors on disease transmission in these regions.

**Methods:**

We used the Xinjiang Uygur Autonomous Region as the study area to investigate the impact of environmental factors on COVID-19 variation using a dynamic disease model. Given the special control and prevention strategies against COVID-19 in Xinjiang, the focus was on social and environmental factors, including population mobility, quarantine rates, and return rates. The model performance was evaluated using the statistical metrics of correlation coefficient (CC), normalized absolute error (NAE), root mean square error (RMSE), and distance between the simulation and observation (DISO) indices. Scenario analyses of COVID-19 in Xinjiang encompassed three aspects: different population mobilities, quarantine rates, and return rates.

**Results:**

The results suggest that the established dynamic disease model can accurately simulate and predict COVID-19 variations with high accuracy. This model had a CC value of 0.96 and a DISO value of less than 0.35. According to the scenario analysis results, population mobilities have a large impact on COVID-19 variations, with quarantine rates having a stronger impact than return rates.

**Conclusion:**

These results provide scientific insight into the control and prevention of COVID-19 in Xinjiang, considering the influence of social and environmental factors on COVID-19 variation. The control and prevention strategies for COVID-19 examined in this study may also be useful for the control of other infectious diseases, especially in low-income regions that are bordered by many countries.

## 1 Introduction

The coronavirus disease (COVID-19) pandemic, caused by severe acute respiratory syndrome coronavirus 2 (SARS-CoV-2), has been a global public health problem since 2020 and has changed all aspects of society and natural ecosystems. As of August 30, 2023, more than 770 million confirmed cases and more than 6.95 million deaths due to COVID-19 were reported, according to the World Health Organization.^1^ The pandemic has adversely affected regional and global economic growth, and post-COVID-19 economic recovery has been slow (1–3). Global levels of nitrous oxide and wetland methane have changed during the COVID-19 lockdown (4–6). Moreover, the COVID-19 pandemic is likely to alter the world order (7).

To control the COVID-19 pandemic, numerous measures have been employed in the last 3 years, such as non-pharmaceutical interventions (NPIs) (e.g., lockdown, limiting public gatherings, physical distancing, and quarantine) and universal mass vaccination (8–11). Accurate simulation and prediction of COVID-19 using mathematical models can provide scientific guidance for the application of reasonable interventions (12–16). Future variations in the COVID-19 pandemic in 88 countries have been explored using the innovation method of Yi Hua Jie Mu based on the Koppen–Geiger climate classification (17).

Highly accurate simulation and prediction of COVID-19 have been performed using the distance between indices of simulation and observation (DISO) (18, 19) in Guangzhou, mainland China, and Kazakhstan using dynamic epidemic models (20–24). Cross-border transmission of COVID-19 can have more adverse effects in undeveloped regions (e.g., Xinjiang) than in developed areas. Xinjiang has the largest area among all provinces in China and borders eight countries. With the development of the One Belt and One Road, the movement of people between Xinjiang and other countries has increased, complicating the control of COVID-19. Thus, the simulation and prediction of COVID-19 based on mathematical models will play a key role in the precise control and prevention of this pandemic and provide important insights for the prevention and control of other infectious diseases in the future.

Therefore, in this study, we focused on Xinjiang using a dynamic epidemic model to simulate and predict COVID-19 pandemic variations and project dynamic changes under different scenarios. In the second section below, a dynamic epidemic model is established according to COVID-19 variations in Xinjiang, and different scenarios are set based on different population mobilities and quarantine measures. The simulation and prediction results are displayed in the third section. Moreover, to investigate the dynamic variations of COVID-19 in Xinjiang that are sensitive to key parameters, we set different scenarios, and a brief conclusion is provided in the last section.

## 2 Constructing a susceptible-exposed- asymptomatic-infectious-recovered epidemic model of COVID-19 in Xinjiang

**Figure 1:**
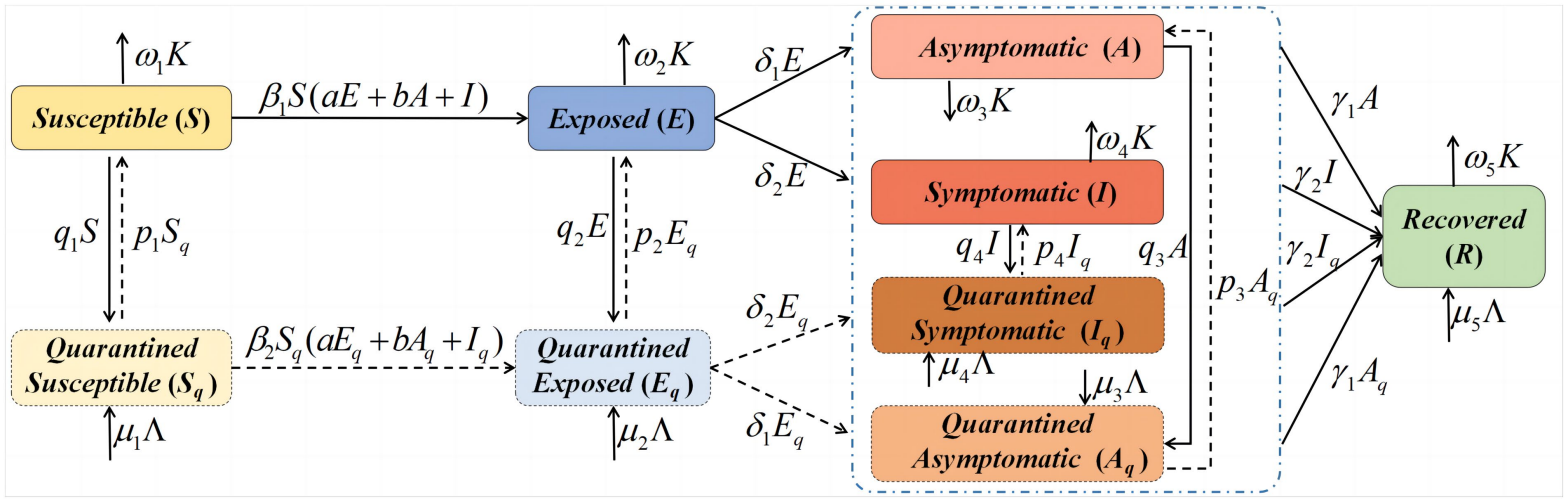
Flowchart of the SEAIR model for coronavirus disease (COVID-19) in Xinjiang. SEAIR, Susceptible-exposed-asymptomatic-infectious-recovered.

Xinjiang has experienced three waves of COVID-19 since 2019. The dynamic zero COVID-19 strategy, which has been employed throughout the pandemic, has been effective in protecting the local population. Given the COVID-19 variations in Xinjiang, a dynamic epidemic model was constructed as follows:

The population was divided into five groups: susceptible (*S*), exposed (*E*), asymptomatic (*A*), symptomatic (*I*), and recovered (*R*). Considering quarantine measures, the corresponding quarantined populations were defined as quarantined susceptible populations (*S_q_*), quarantined exposed populations (*E_q_*), quarantined asymptomatic populations (*A_q_*), and quarantined symptomatic populations (*I_q_*). *K* is the output population at time *t* and Λ denotes the input population at time *t*. COVID-19 has a bilinear incidence rate, with *β*_1_ for the unquarantined population and *β*_2_ for the quarantined population; *a* is the fraction of the transmission rate for *E*, and *b* is the fraction of transmission rate for *A*. *δ*_1_ and *δ*_2_ are the transmission rates from *E* to *A* and *I*, respectively. *Γ*_1_ and *γ*_2_ are the recovery rates for *A* and *I*, respectively. *Q_i_* (*i* = 1, 2, 3, 4) is the quarantine rate of *S*, *E*, *A*, and *I* populations, respectively, and *p_i_* (*i* = 1, 2, 3, 4) is the release rate of *S_q_*, *E_q_*, *A_q_*, and *I_q_* populations, respectively. Details of the other parameters of the dynamic SEAIR model are provided in Table 1. A flowchart of the dynamic variations of COVID-19 in Xinjiang is shown in Figure 1. The corresponding dynamic SEAIR epidemic model was constructed, as shown in the model (2.1).

**Table 1:** Parameter estimations of the SEAIR model for COVID-19 in Xinjiang.

Parameter	Definitions	Estimated value	Source
β_1_	Transmission incidence rate of unquarantined individuals	4.95 × 10^−8^	Estimated
β_2_	Transmission incidence rate of quarantined individuals	3.14 × 10^−9^	Estimated
*a*	The fraction of transmission incidence rate for *E* class	0.4	Assumed
*b*	The fraction of transmission incidence rate for *A* class	0.95	Assumed
*δ*_1_	Transmission rate of individuals from *E* class to *A* class	0.29	Estimated
*δ_2_*	Transmission rate of individuals from *E* class to *I* class	0.0082	Estimated
*γ*_1_	The recovery rate of asymptomatic class	1/4	Assumed
*γ_2_*	The recovery rate of symptomatic class	1/14	Assumed
*q*_1_	Quarantined rate of *S* class	0.15	Estimated
*q*_2_	Quarantined rate of *E-class*	0.15	Estimated
*q*_3_	Quarantined rate of *A-class*	0.22	Estimated
*q*_4_	Quarantined rate of *I* class	0.26	Estimated
*p*_1_	The return rate of *S_q_* class	0.015	Estimated
*p*_2_	The return rate of *E_q_* class	0.01	Estimated
*p*_3_	The return rate of *A_q_* class	0.01	Estimated
*p*_4_	The return rate of *I_q_* class	0.001	Estimated
K	The Output population at time *t*	-	Estimated
Λ	The Iutput population at time *t*	-	Estimated
Initial values	Definitions	Estimated value	Source
N(0)	Initial total population	2.589 × 10^7^	Data
S(0)	Initial susceptible population	1.5 × 10^7^	Estimated
E(0)	Initial exposed population	300	Estimated
A(0)	Initial asymptomatic population	600	Estimated
I(0)	Initial symptomatic population	5	Estimated
*S_q_*(0)	Initial quarantined susceptible population	1.0887 × 10^7^	Estimated
*E_q_*(0)	Initial quarantined exposed population	180	Estimated
*A_q_*(0)	Initial quarantined asymptomatic population	300	Estimated
*I_q_*(0)	Initial quarantined symptomatic population	4	Estimated
R(0)	Initial recovered population	1,005	Data

After constructing the SEAIR model, the parameters in Table 1 were established by the least-squares method using COVID-19 data from Xinjiang from August 6 to September 27, 2022 (data from National Health Commission of the People’s Republic of China; http://www.nhc.gov.cn/). Using the established parameters, the cumulative cases, cumulative asymptomatic cases, daily new cases, and daily new asymptomatic cases will be simulated in the next section. To quantify the simulation performance of the model (2.1), some statistical metrics were employed (21), including the correlation coefficient (CC), absolute error (AE), root mean square error (RMSE), and DISO (18, 19, 23). They are expressed as follows:


$$CC=\frac{\sum_{i=1}^{n}\left(a_{i}-\bar{a}\right)\left(b_{i}-\bar{b}\right)}{\sqrt{\sum_{i=1}^{n}{\left(a_{i}-\bar{a}\right)}^{2}}\sqrt{\sum_{i=1}^{n}{\left(b_{i}-\bar{b}\right)}^{2}}}$$

$$AE=\frac{1}{n}\sum_{i=1}^{n}\left(b_{i}-a_{i}\right)$$

$$\mathrm{RMSE}=\sqrt{\frac{1}{n}\sum_{i=1}^{n}\left(b_{i}-a_{i}\right)}$$

$$\mathrm{DISO}=\sqrt{{\left(CC-1\right)}^{2}+NAE^{2}+\mathrm{NRMS}E^{2}}$$

where *a_i_* and *b_i_* (*i* = 1, 2, *…*, *n*) represent the observed and simulated data, respectively. *NAE* and *NRMSE* are normalized by the average values of the observed time series.


(2.1) $$\left\{ \begin{array}{c} \begin{array}{l} \frac{dS\left(t\right)}{dt}=-\beta_{1}S\left(t\right)\left(aE\left(t\right)+\mathrm{bA}\left(t\right)+I\left(t\right)\right)\\ \;q_{1}S\left(t\right)+p_{1}S_{q}\left(t\right)-\omega_{1}K \end{array}\\ \begin{array}{l} \frac{dE\left(t\right)}{dt}=\beta_{1}S\left(t\right)\left(aE\left(t\right)+\mathrm{bA}\left(t\right)+I\left(t\right)\right)-\\ \left(\delta_{1}+\delta_{2}\right)E\left(t\right)-q_{2}E\left(t\right)+p_{2}E_{q}\left(t\right)-\omega_{2}K, \end{array}\\ \begin{array}{l} \frac{dA\left(t\right)}{dt}=\delta_{1}E\left(t\right)-\gamma_{1}A\left(t\right)-\\ \;q_{3}A\left(t\right)+p_{3}A_{q}\left(t\right)-\omega_{3}K, \end{array}\\ \begin{array}{l} \frac{dI\left(t\right)}{dt}=\delta_{2}E\left(t\right)-\gamma_{2}A\left(t\right)-\\ \;q_{4}I\left(t\right)+p_{4}I_{q}\left(t\right)-\omega_{4}K, \end{array}\\ \frac{dS_{q}\left(t\right)}{dt}=\mu_{1}\Lambda -\beta_{2}S_{q}\left(t\right)\left(\begin{array}{l} aE_{q}\left(t\right)+\\ bA_{q}\left(t\right)+\\ I_{q}\left(t\right) \end{array}\right)+q_{1}S\left(t\right)-p_{1}S_{q}\left(t\right),\\ \begin{array}{l} \frac{dE_{q}\left(t\right)}{dt}=\mu_{2}\Lambda +\beta_{2}S_{q}\left(t\right)\left(\begin{array}{l} aE_{q}\left(t\right)+\\ bA_{q}\left(t\right)+\\ I_{q}\left(t\right) \end{array}\right)-\left(\delta_{1}+\delta_{2}\right)E_{q}\left(t\right)+\\ \;q_{2}E\left(t\right)+p_{2}E_{q}\left(t\right), \end{array}\\ \begin{array}{l} \frac{dA_{q}\left(t\right)}{dt}=\mu_{3}\Lambda +\delta_{1}E_{q}\left(t\right)-\\ \;\gamma_{1}A_{q}\left(t\right)+q_{3}A\left(t\right)-p_{3}A_{q}\left(t\right), \end{array}\\ \begin{array}{l} \frac{dI_{q}\left(t\right)}{dt}=\mu_{4}\Lambda +\delta_{2}E_{q}\left(t\right)-\\ \;\gamma_{2}I_{q}\left(t\right)+q_{4}A\left(t\right)-p_{4}I_{q}\left(t\right), \end{array}\\ \begin{array}{l} \frac{dR\left(t\right)}{dt}=\mu_{5}\Lambda +\gamma_{1}\left(A\left(t\right)+A_{q}\left(t\right)\right)+\\ \;\gamma_{2}\left(I\left(t\right)+I_{q}\left(t\right)\right)-\omega_{5}K\text{,} \end{array} \end{array}\right.$$

To investigate the dynamic variations of COVID-19 in Xinjiang that are sensitive to key parameters, we set different scenarios with different population mobilities, quarantine rates, and return rates. All the simulations and predictions were obtained by the Mathematica 9.0 software.

## 3 Results

In this section, the COVID-19 variations in Xinjiang were simulated and predicted using model (2.1). Simulation and prediction accuracies were quantified using CC, AE, RMSE, and DISO. Different scenario analyses were performed to investigate the sensitivities of COVID-19 variations to key parameters.

### 3.1 Simulation and prediction of COVID-19 in Xinjiang based on model (2.1)

**Figure 2:**
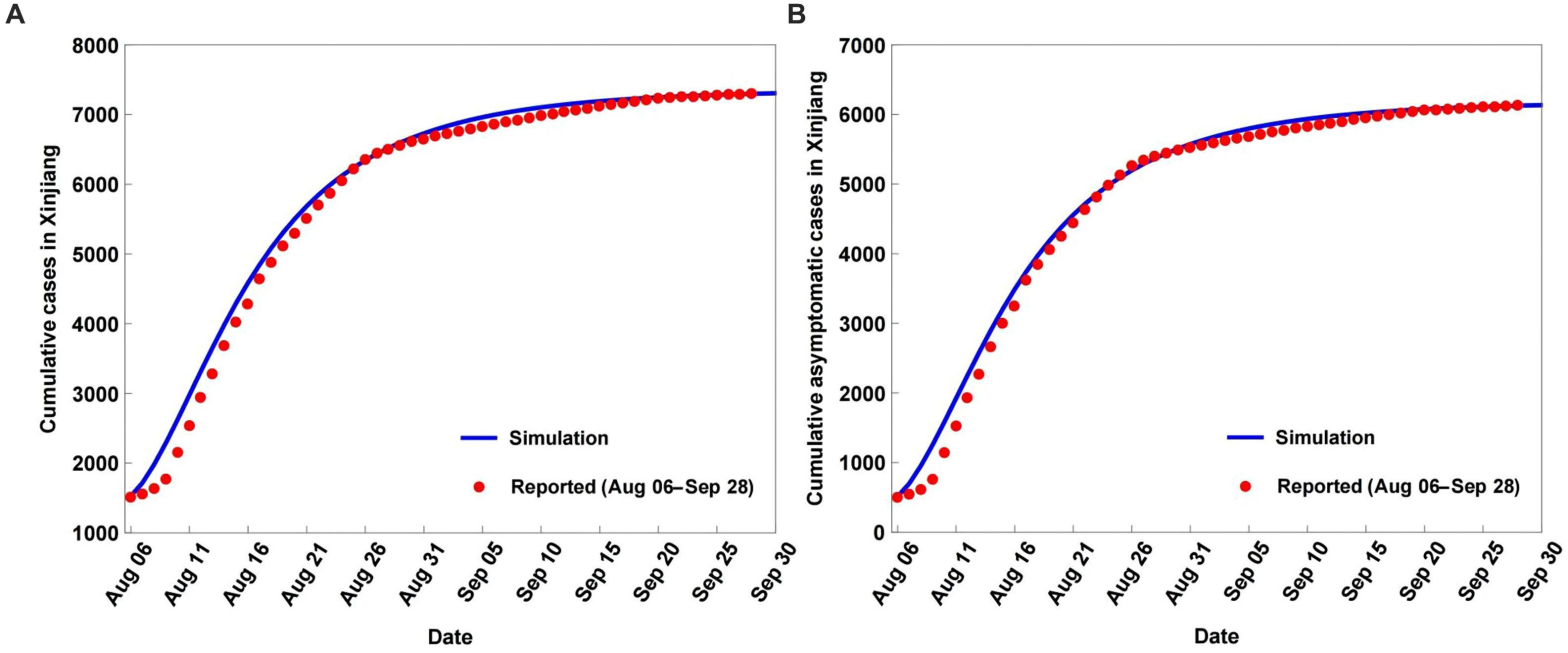
Simulation and prediction results of the cumulative cases **(A)** and cumulative asymptomatic cases **(B)** of coronavirus disease (COVID-19) in Xinjiang from August 6 to September 28, 2022.

**Figure 3:**
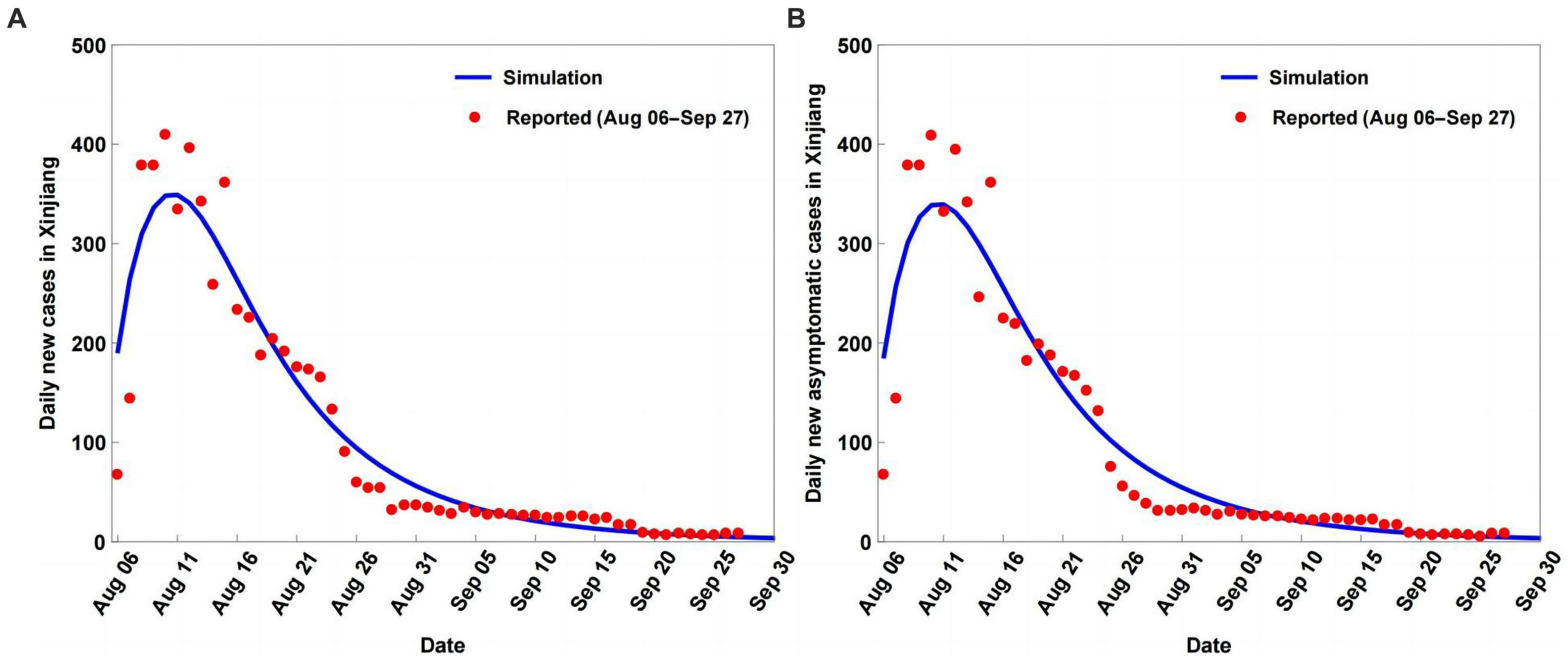
Simulation and prediction results of the daily new cases **(A)** and daily new asymptomatic cases **(B)** of coronavirus disease (COVID-19) in Xinjiang from August 6 to September 28, 2022.

The COVID-19 variations were simulated and predicted based on model (2.1) and the parameters in Table 1, as shown in Figures 2, 3. The model (2.1) captures the cumulative case variations and cumulative asymptomatic case variations well (Figure 2). The CC values for the simulated cumulative cases and simulated cumulative asymptomatic cases against the OBS were both 0.9988 (Table 2). The simulated results overestimated the OBS, with RE values of 0.0339 and 0.0520, respectively. The RMSE values were 0.0295 and 0.0314, respectively, and DISO values were 0.0449 and 0.0607, respectively.

**Table 2:** Evaluation results of the simulation and prediction of daily new confirmed cases and cumulative confirmed COVID-19 cases in Xinjiang.

Case	CC	Name	RMSE	DISO
Cumulative	0.9988	0.0339	0.0295	0.0450
Cumulative asymptomatic cases	0.9988	0.0520	0.0314	0.0607
Daily new cases	0.9610	0.0060	0.3180	0.3204
Daily new asymptomatic cases	0.9589	0.0363	0.3376	0.3420

CC, Correlation coefficient; COVID-19, Coronavirus disease; DISO, Distance between the simulation and observation; NAE, Normalized absolute error; and RMSE, Root mean square error.

For the simulated daily new cases and simulated daily new asymptomatic cases, model (2.1) captured the observed daily new cases and observed daily new asymptomatic case variations, with underestimation of the peak values and slight overestimation of the smallest values (Figure 3). The simulated results of the daily new cases and daily new asymptomatic cases had lower accuracy than the simulated cumulative cases and cumulative asymptomatic cases (Table 2). The CC values for the simulated daily new cases and simulated daily new asymptomatic cases against the OBS were 0.9610 and 0.9589, respectively (Table 2), while RMSE values were 0.3180 and 0.3376, respectively. The corresponding DISO values, which were used to examine the overall performance of model (2.1), were 0.3204 and 0.3420.

### 3.2 Scenario analysis of COVID-19 in Xinjiang with different key parameters

Given that the transmission of infectious diseases is generally influenced by some key factors (24), the impact of these key parameters in the model (2.1) on the dynamic variations of COVID-19 in Xinjiang should be investigated. In this section, scenario analyses of COVID-19 in Xinjiang comprise three aspects: different population mobilities, quarantine rates, and return rates. For comparison with the scenario results, the COVID-19 variations with the established parameters in Section 3.1 are defined as the baseline results.

#### 3.2.1 Scenario analyses of COVID-19 in Xinjiang with different population mobilities

In this section, we identify the impacts of the input population Λ and output population *K* on the COVID-19 variations in Xinjiang. The size of the population is controlled by changing the numbers of Λ and *K*, that is, the percentage parameter values (*μ* and *ω*) of different populations are changed.

For the changed numbers of the input population Λ and output population *K*, the scenarios are set as follows: (1) fix the input population Λ number and change (i.e., increasing and decreasing) the output population *K* number; (2) fix the output population *K* number and change (i.e., increasing and decreasing) the output population Λ number.

**Figure 4:**
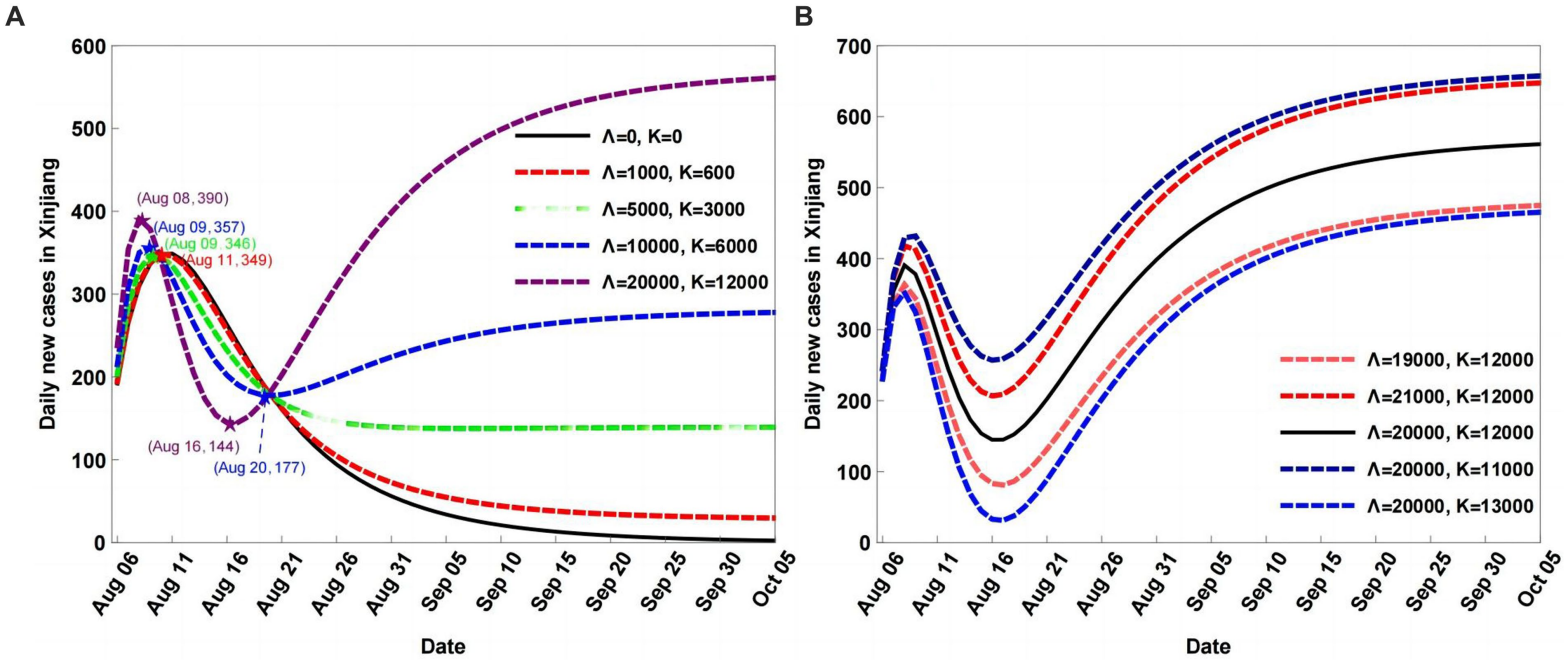
Scenario analyses of daily new cases with different input and output population numbers (A), fixed the input population Λ number and changing the output population K number;fixed the output population K number and changing the output population Λ number (B).

By increasing the number of Λ and fixing the number of *K*, the local maximum daily new cases increase and the corresponding time is delayed compared to the baseline. Moreover, the number of daily new cases increases with time (Figure 4). When Λ is decreased and *K* is fixed, the local maximum daily new cases reduce at an earlier time than that for the baseline. Over time, a further decrease in the number of daily new cases is observed.

When the number of Λ is fixed at baseline and the *K* number is increased, the local maximum daily new cases are reduced at an earlier time. Moreover, the degree of decrease in the daily new cases is larger than when the number of Λ is decreased and the number of *K* is fixed (Figure 4). When the number of Λ is fixed and that of *K* decreases, the daily new cases increase compared to the baseline, which indicates that the higher the population number, the higher the number of infected cases.

#### 3.2.2 Scenario analysis of COVID-19 in Xinjiang with different percentage rates of Λ and *K* for different populations

**Figure 5:**
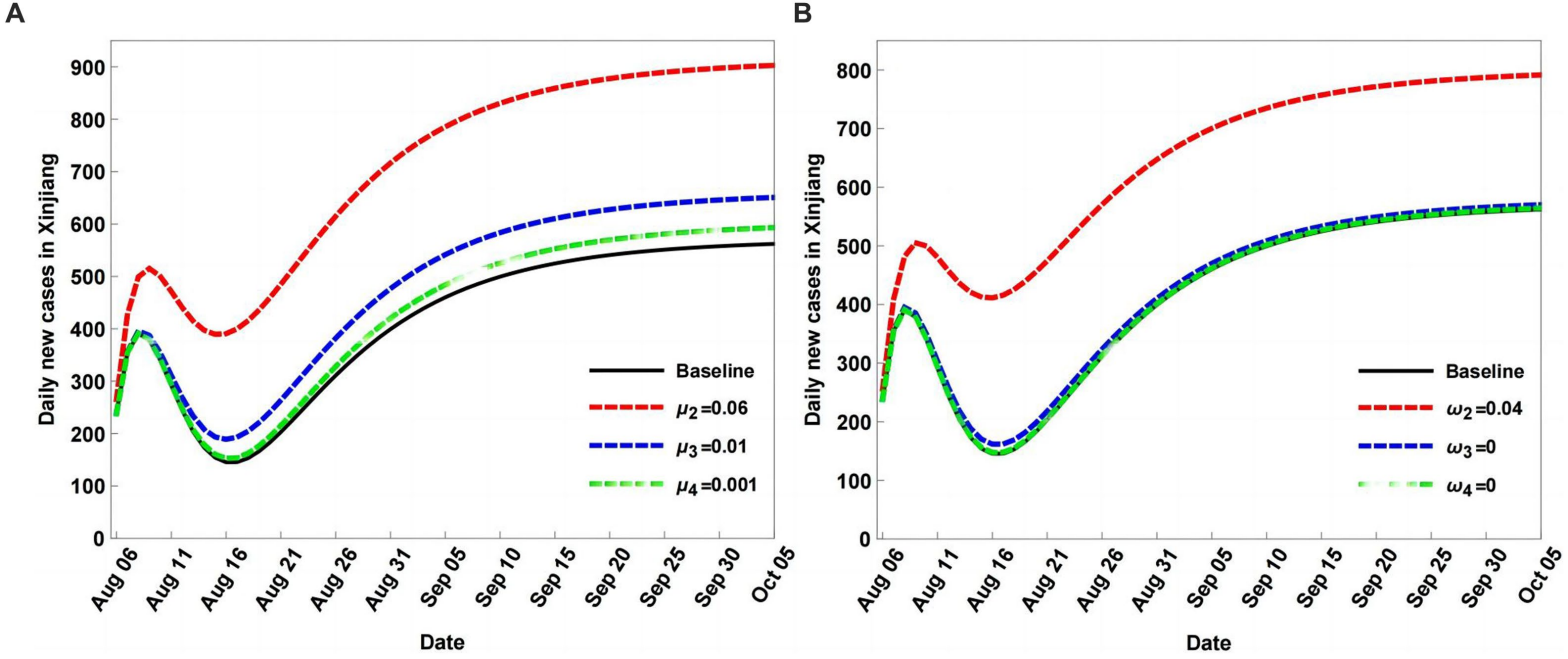
Scenario analyses of daily new cases of coronavirus disease (COVID-19) with different percentage parameters μ and ω values **(B)**. In **(A)**, the baseline with $\mu_{1}=1-\sum_{i=2}^{5}$ *μ_i_* = 0.9488, *μ*_2_ = 0.05, *μ*_3_ = 0.001, *μ*_4_ = 0.0001, *μ*_5_ = 0.0001; In **(B)**, the baseline with same values as in **(A)**, but for ω.

During the COVID-19 pandemic, population mobility increases when the exposed population *E* is added, including the asymptomatic population. Therefore, the percentages of different populations in the input population Λ and output population *K* have serious impacts on COVID-19 transmission. In this section, *μ_i_* (*i* = 1, 2, 3, 4) and *ω_i_* (*i* = 1, 2, 3, 4) represent the percentage rates of *S*, *E*, *A*, and *I* for Λ and *K*, respectively. Assuming Λ = 20,000 and *K* = 12,000 at baseline, when the percentage values of different populations for *K* are fixed, changing the percentage *μ* and *ω* values results in the variation of daily new cases (Figure 5). Figure 5A shows the variations in daily new cases, with the percentage parameter *μ* larger than the baseline. When the percentage rates *μ_i_* (*i* = 2, 3, 4) of *E*, *A*, and *I* for Λ are increased, the daily new cases also increase compared to baseline.

Among *μ*_2_, *μ*_3_, and *μ*_4_, the percentage *μ*_2_ of *E* has the largest impact on COVID-19 transmission. A larger percentage *μ*_2_ of Λ can result in a larger infected population. When the percentage values of different populations for Λ are fixed and *ω_i_* (*i* = 2, 3, 4) are decreased, the daily new cases increase compared to baseline (Figure 5B). In addition, the percentage of the exposed population *E* is suggested to have the largest impact compared with the percentage of the other populations.

#### 3.2.3 Scenario analysis of COVID-19 in Xinjiang with different quarantined rates

In the past 3 years, the dynamic zero COVID-19 strategy has been employed in Xinjiang to prevent and control COVID-19. Therefore, adjusting quarantine strategies has a significant impact on COVID-19 transmission. In this section, different scenarios are explored by adjusting the strength of the quarantine strategy compared to the baseline, assuming no input or output populations.

**Figure 6:**
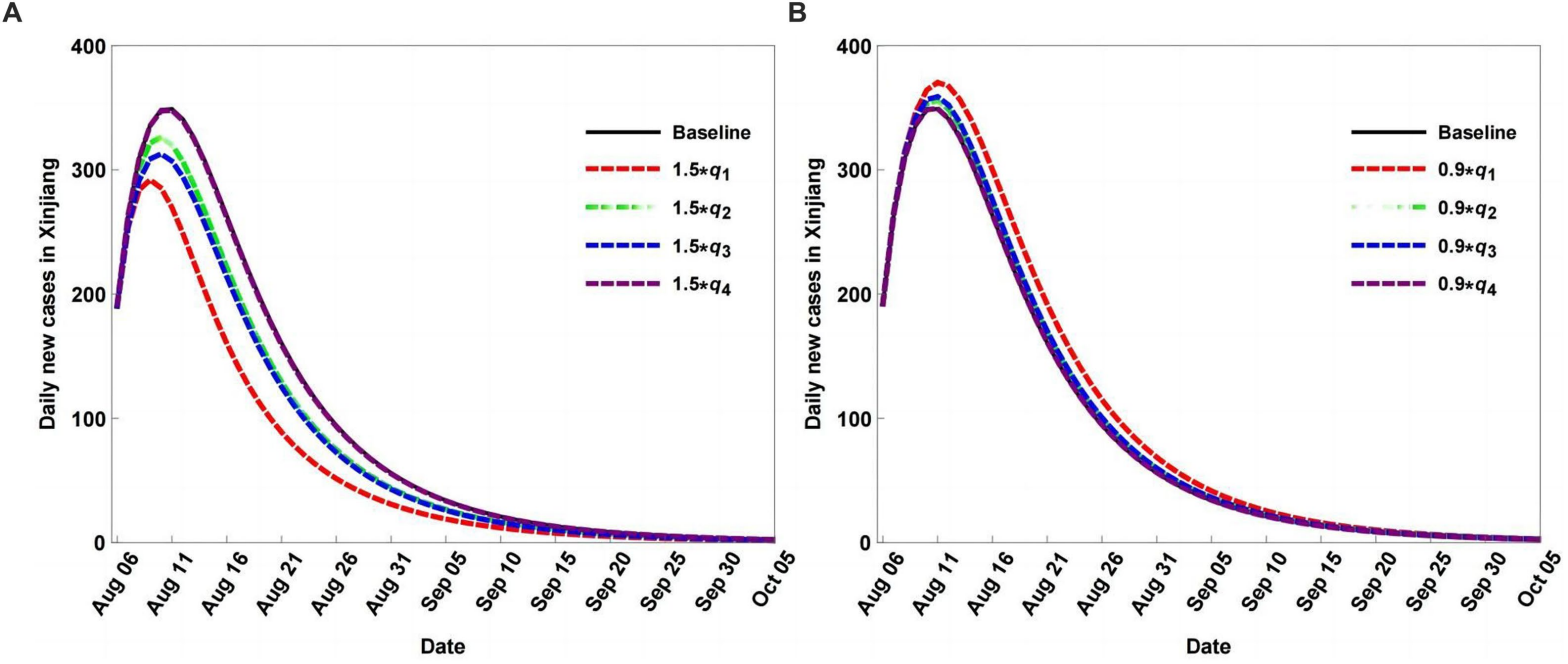
Scenario analysis of daily new cases with different quarantined rates, rates decrease (A), and rates increse (B).

Adjusting quarantine rates can change the peak values and peak value times, as shown in Figure 6. In particular, when the quarantine rates decrease, the peak values reduce and are reached earlier than the baseline (Figure 6A). When the quarantine rates are increased, the peak values also increase, and the peak value times are delayed more than the baseline (Figure 6B). Moreover, the impacts of quarantine on S and A are greater than those on E and I, indicating that quarantine is more effective for S and A than for E and I.

#### 3.2.4 Scenario analysis of COVID-19 in Xinjiang with different return rates

**Figure 7:**
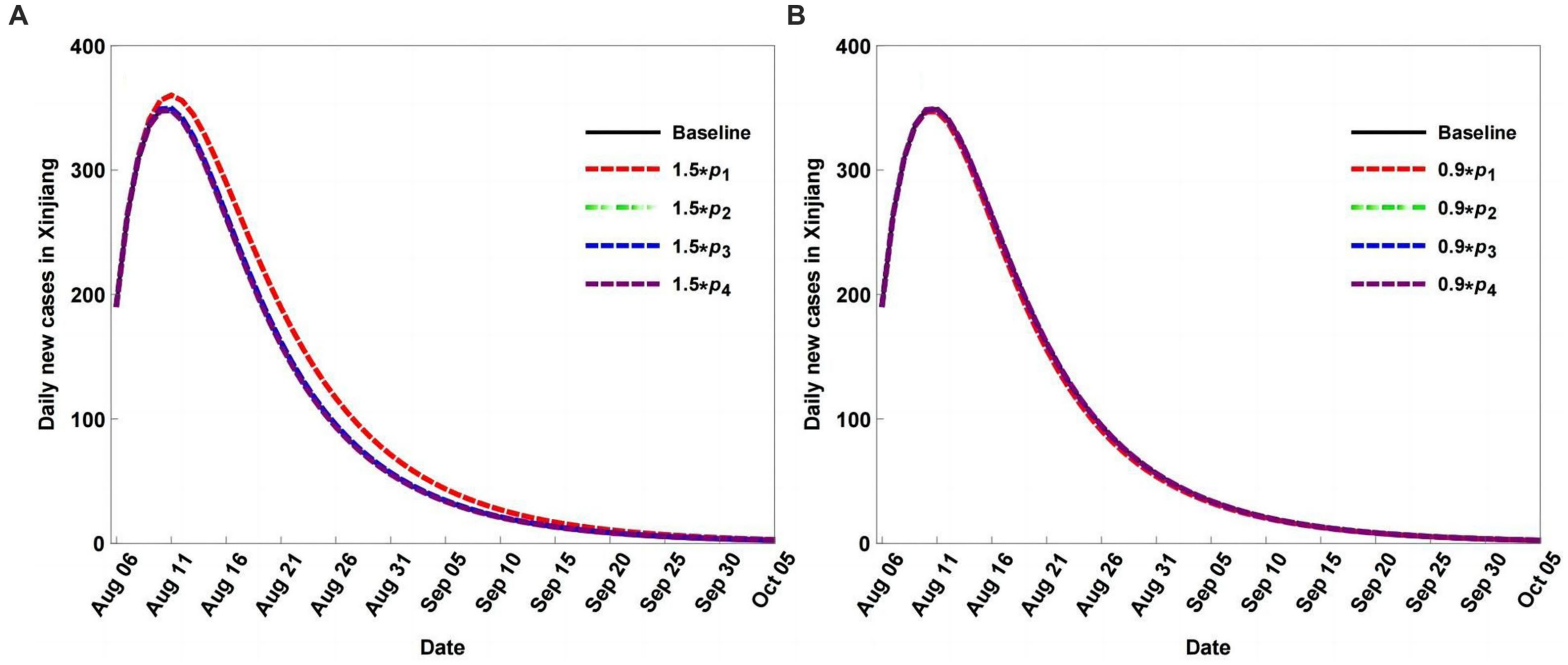
Scenario analysis of daily new cases with different return rates, rates decrease (A), and rates increase (B).

Given the dynamic zero COVID-19 strategy, the quarantine strategy is adjusted, allowing the quarantined population to return to the non-quarantined population. Therefore, we set different return rates to explore their impact on COVID-19 transmission with non-population mobility. Figure 7 shows that return rate variations have a weak impact on disease transmission, with small changes in the peak values of daily new cases. Compared with the quarantine rates, the influence of the return rates was smaller (Figures 6, 7).

## 4 Discussion

During the past years, how the environmental factors (i.e., mainly including natural factors and social factors) impacting the COVID-19 variations has obtained lots of attention. For the natural factors (e.g., climate factors), our previous work suggested that temperate and cold climate regions had a larger transmission rate than arid and tropical climate regions (17). The daily death counts of COVID-19 were negatively influenced by the absolute humidity (25). For the social factors, the large total population number with high population density can cause large infected cases of COVID-19 in large cities (26). To boost vaccine effectiveness, infection control measures can minimize the COVID-19 infection for the at-risk populations (27).

Many factors have a significant impact on the incidence, transmission, and outbreak of human infectious diseases. The One Health concept seeks to integrate all the elements of environmental, animal, and human health and track the entire disease process from incidence to extinction. Environmental, animal, and human health interact closely and influence disease occurrence.

Generally, environmental factors include natural (e.g., temperature, precipitation, and wind) and social environmental elements (e.g., population mobility, population density, and government measures). As a zoonotic disease, the outbreak and transmission of COVID-19 are influenced closely by environmental and animal factors (6, 16, 17). Understanding the impact of environmental factors on COVID-19 plays a key role in controlling and preventing its transmission. The NPIs employed by the government and local population comprise adjustments to social and environmental factors.

Numerous studies have focused on the influence of social and environmental factors on COVID-19 (9–11, 15). The roadmap policy can successfully offset the increased transmission resulting from the lifting of NPIs (11). The early detection and isolation of cases prevented more infections than travel restrictions and contact reduction, and the combination of NPIs achieved the strongest and most rapid effect (9). This study suggests that strict management of population movement plays a significant role in reducing the risk of COVID-19 transmission, which is consistent with our previous results (21, 22). However, Walker et al. (3) pointed out that increasing the income of low- and middle-income countries should be a global priority for controlling and preventing disease transmission.

In model (2.1), most parameters are estimated by the least square method using the limited real-world data, which may result in some uncertainties about the simulation and prediction results. However, the limited real-world data are restricted by the data management policy. Another limitation of this study is the constant value of the transmission incidence rate. The transmission incidence rate is mostly determined by the contact rate and the probability of transmission per contact. To address these limitations, we wish more special real-world data can be obtained in the future.

Other interesting topics related to COVID-19 in Xinjiang should be considered. For example, the impact of vaccination on disease transmission, the age structure of the infected population, and multiple wave simulation can be explored for in-depth analysis. These topics will be studied in the future when the relevant datasets become available.

## 5 Conclusion

In this study, the impacts of major social environmental factors, particularly population mobility, quarantine measures, and return rates, on COVID-19 in Xinjiang have been analyzed comprehensively using a dynamic epidemic model.

The major conclusions are as follows:

1. The established dynamic SEAIR model can capture COVID-19 variations in Xinjiang with high accuracy. The SEAIR model weakly overestimates cumulative and new daily cases, with an AE of approximately zero. The CC values between the OBS and simulated data were greater than 0.95. The DISO values were smaller than 0.5, indicating the high overall performance of the model.
2. For population mobility, when the input population Λ is increased with the output population K fixed, the infected population increases, and the disease transmission is strengthened. When the output population K is decreased with the input population Λ fixed, the infected population also increases. These findings suggest that decreasing the input population and increasing the output population are useful for controlling disease transmission.
3. The percentages of different populations in the input and output populations play important roles in controlling and preventing diseases. Decreasing the percentages of S and E in the input population can reduce the infected population.
4. When there is no external population mobility, quarantine measures, especially for susceptible and asymptomatic populations, have a large impact on disease transmission. In other words, the return rates of different populations have similar impacts on disease transmission as quarantine rates do.

The above analyses comprehensively explored the impact of major social and environmental factors on disease transmission. These results provide insight into the control and prevention of COVID-19 in Xinjiang and can be useful in managing outbreaks of other infectious diseases.

## Data availability statement

The original contributions presented in the study are included in the article/supplementary material; further inquiries can be directed to the corresponding author.

## Author contributions

RF: Conceptualization, Writing – original draft, Writing – review & editing. WL: Conceptualization, Visualization, Writing – review & editing. SW: Data curation, Writing – original draft. JZ: Data curation, Investigation, Writing – original draft. QC: Data curation, Writing – original draft. ZH: Writing – original draft, Writing – review & editing. LZ: Conceptualization, Data curation, Investigation, Writing – original draft, Writing – review & editing. FW: Writing – original draft, Writing – review & editing.

## Footnotes

^1^https://covid19.who.int/
